# Identification of zinc and Zur-regulated genes in *Corynebacterium diphtheriae*

**DOI:** 10.1371/journal.pone.0221711

**Published:** 2019-08-27

**Authors:** Eric D. Peng, Michael P. Schmitt

**Affiliations:** Laboratory of Respiratory and Special Pathogens, Division of Bacterial, Parasitic, and Allergenic Products, Center for Biologics Evaluation and Research, Food and Drug Administration,Silver Spring, MD, United States of America; Universite Paris-Sud, FRANCE

## Abstract

*Corynebacterium diphtheriae* is a Gram-positive bacterial pathogen and the causative agent of diphtheria, a severe disease of the upper respiratory tract of humans. Factors required for *C*. *diphtheriae* to survive in the human host are not well defined, but likely include the acquisition of essential metals such as zinc. In *C*. *diphtheriae*, zinc-responsive global gene regulation is controlled by the Zinc Uptake Regulator (Zur), a member of the Fur-family of transcriptional regulators. In this study, we use transcriptomics to identify zinc-regulated genes in *C*. *diphtheriae* by comparing gene expression of a wild-type strain grown without and with zinc supplementation. Zur-regulated genes were identified by comparing wild-type gene expression with that of an isogenic *zur* mutant. We observed zinc repression of several putative surface proteins, the heme efflux system *hrtBA*, various ABC transporters, and the non-ribosomal peptide synthetase/polyketide synthase cluster *sidAB*. Furthermore, increased gene expression in response to zinc was observed for the alcohol dehydrogenase, *adhA*. Zinc and Zur regulation were confirmed for several genes by complementing the *zur* deletion and subsequent RT-qPCR analysis. We used MEME to predict Zur binding sites within the promoter regions of zinc- and Zur-regulated genes, and verified Zur binding by electrophoretic mobility shift assays. Additionally, we characterized *cztA* (*dip1101*), which encodes a putative cobalt/zinc/cadmium efflux family protein. Deletion of *cztA* results in increased sensitivity to zinc, but not to cobalt or cadmium. This study advances our knowledge of changes to Zur-dependent global gene expression in response to zinc in *C*. *diphtheriae*. The identification of zinc-regulated ABC transporters herein will facilitate future studies to characterize zinc transport in *C*. *diphtheriae*.

## Introduction

*Corynebacterium diphtheriae* is the causative agent of the respiratory disease diphtheria, an acute illness that results in inflammation of the upper respiratory tract and causes systemic organ damage due to the production of the potent exotoxin, diphtheria toxin [1].While little is known regarding the factors required for *C*. *diphtheriae* to survive and colonize the human respiratory tract, the acquisition of essential nutrients, including zinc, is likely critical for the pathogen to cause disease. Zinc is an essential trace element with structural and catalytic roles in numerous proteins, and it is estimated that 5–6% of bacterial proteins require zinc as a co-factor [2]. The acquisition of the metal is a challenge to invading bacterial pathogens due to host-imposed restriction. Evidence from animal models suggest that free zinc is abundant in the airway epithelium [3], but limited in plasma [4]. Furthermore, S100 proteins, such as calprotectin, and other host mechanisms sequester multiple metals, including zinc, in response to bacterial infection [5–7]. Therefore, *C*. *diphtheriae*, like other bacterial pathogens, likely requires specific transport systems to acquire sufficient zinc for survival. Zinc import in *C*. *diphtheriae* is poorly understood. Studies of *C*. *diphtheriae* in which several genes encoding putative zinc transporters were deleted from the chromosome showed no defect in the ability to grow in zinc-depleted medium, suggesting that multiple systems likely contribute to the uptake of extracellular zinc [8].

In addition to limited availability within the host, excess zinc can also be toxic to cells. Zinc intoxication is thought to occur when high levels of intracellular zinc cause mis-metalation by displacing other metal ions in enzymes [9]. As part of the innate immune system, zinc toxicity is used by macrophages to eliminate bacterial pathogens [10]. To avoid zinc toxicity, numerous bacteria encode mechanisms for regulating both the import and export of zinc, which are needed for maintaining zinc homeostasis. Almost all bacterial pathogens encode transcriptional regulators that control expression of zinc transport systems, of which the best characterized protein is the Zinc Uptake Regulator, Zur, a member of the Fur family of DNA-binding transcriptional regulators. The Zur protein is widely distributed in bacteria including most *Corynebacterium* species and the related genus *Mycobacterium* [11]. Intracellular zinc levels are monitored by direct binding of the metal ion to Zur, which results in a conformational change in the protein that allows Zur to bind to target sequences on the DNA to control gene transcription [12]. A Zur homolog was previously identified in *C*. *diphtheriae* and deletion of the *zur* gene resulted in the de-repression of the zinc-regulated genes *troA* (*dip0438*), *zrg* (*dip1486)* and *cmrA* (*dip2325)*, thus confirming the role of Zur in zinc-regulated gene expression in *C*. *diphtheriae* [13]. More recently, Zur was shown to regulate *iutE* (*dip0173*), which is predicted to encode a substrate binding protein that may be associated with manganese or zinc transport [8].

In this study, we use a gene expression array to identify new targets of zinc and Zur regulation in *C*. *diphtheriae*. We identified 37 genes whose transcription is affected by both zinc and Zur, including those encoding several ABC-type transporters, predicted sortase-anchored surface proteins, and *cztA* (*dip1101*), a putative cation efflux protein that is required for growth of *C*. *diphtheriae* in high zinc environments and may function as a zinc-specific efflux system. Several Zur binding sites were confirmed by the binding of recombinant Zur in electrophoretic mobility shift assays (EMSA). This is the first report to describe zinc-dependent gene regulation and the Zur regulon in the important human pathogen *C*. *diphtheriae* and the findings provide an opportunity for future studies to broaden our understanding of zinc transport systems and zinc homeostasis in *C*. *diphtheriae*.

## Results and discussion

### Characterizing the role of Zur in regulating genes in response to zinc

**Fig 1:**
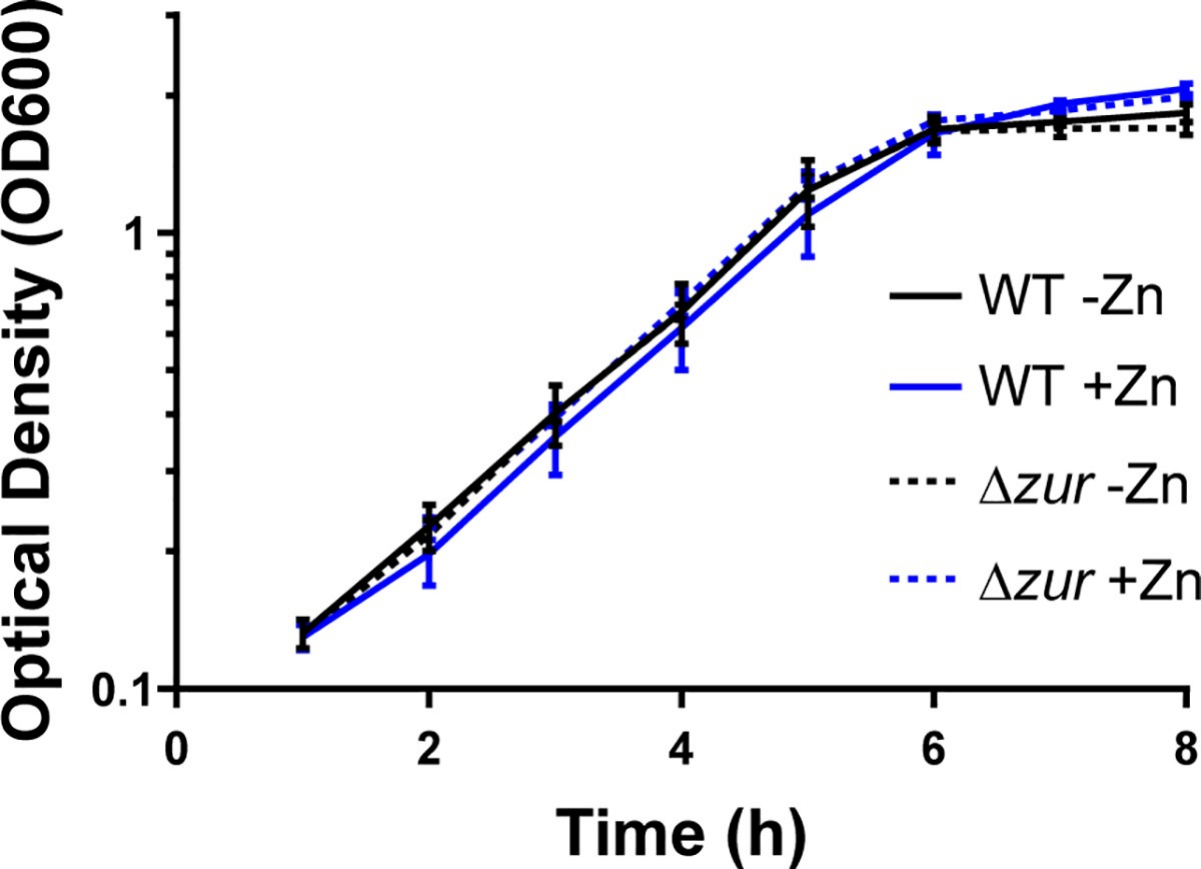
Growth of *C*. *diphtheriae* wild type and *zur* deletion mutant in low-metal media. Wild-type *C*. *diphtheriae* strain 1737 (solid lines) and an isogenic *zur* mutant (dashed lines) were grown in mPGT medium without (black, -Zn) or with (blue, +Zn) zinc chloride at a final concentration of 5 μM. The data are the mean and standard deviation of four biological replicates. Samples were collected for RNA extraction at 4 h with an OD600 of 0.5–0.7.

**Fig 2:**
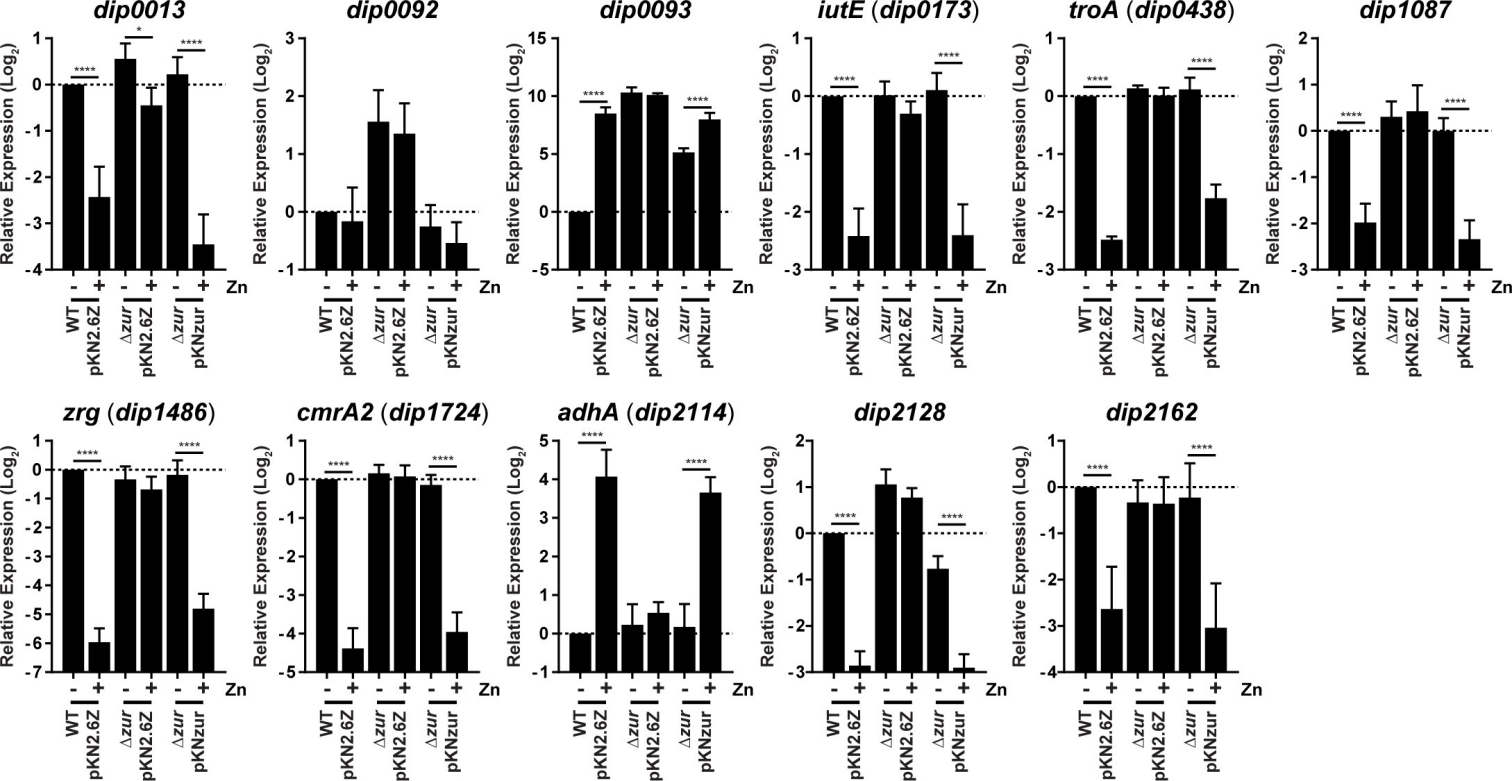
Complementation of the *zur* deletion restores wild-type levels of expression to zinc- and Zur-regulated genes. RNA that was harvested from various *C*. *diphtheriae* strains grown in mPGT without (-) and with (+) 5 μM zinc (Zn) supplementation was used for RT-qPCR. *C*. *diphtheriae* strains include; wild-type *C*. *diphtheriae* carrying vector pKN2.6Z (WTpKN2.6Z) and the *zur* mutant carrying either the vector pKN2.6Z (Δ*zur* pKN2.6Z) or the complementing plasmid containing the *zur* gene, pKNzur (Δ*zur* pKNzur). Relative expression of indicated genes was compared using the ΔΔCq method whereby *gyrB* expression was used for normalization, and the wild-type strain without added zinc [WT (-)] was used for comparison. The data are the mean and standard deviation of three biological replicates; significance was determined by 2way ANOVA, Holm-Sidak’s multiple comparisons test: **** indicates p < 0.0001; * indicates p < 0.05.

To identify genes differentially regulated in response to the availability of zinc, we used the low-metal semi-defined medium, mPGT [14]. Wild-type *C*. *diphtheriae* and an isogenic *zur* mutant [8] were grown in mPGT in the absence and presence of 5 μM zinc chloride. As demonstrated previously, this concentration of zinc is sufficient to repress genes in a Zur-dependent manner [8]. The wild-type strain and the *zur* mutant showed similar growth in the mPGT media, regardless of zinc supplementation (Fig 1). RNA was isolated from bacterial cells during mid-logarithmic phase for gene expression analysis. Table 1 shows genes that were differentially expressed in the wild-type strain grown in the absence and presence of zinc supplementation (WT±Zn), and genes regulated by Zur were identified by comparing the wild type to the *zur* mutant grown only in the presence of added zinc (Δ*zur*:WT). We identified 37 genes in which expression was both zinc and Zur regulated (Table 1). Of the genes identified in our analysis, *zrg*, *cmrA*, *troA* and *iutE* were previously shown to be zinc-regulated in *C*. *diphtheriae* [8, 13]. Some of the notable findings included the identification of several ABC-type metal transporters, as well as two homologous genes, *cmrA* (*dip2325*) and *cmrA2* (*dip1724*), that are predicted to encode large sortase-anchored surface proteins of unknown function. Expression of the *adhA* (*dip2114*) gene, predicted to encode an alcohol dehydrogenase, was activated by Zur in the presence of zinc, suggesting that Zur not only functions as a repressor but also as a zinc-dependent activator of gene expression. The *zur* gene itself was not significantly regulated by zinc in the microarray. The array was validated through RT-qPCR by measuring gene expression of all previously known zinc and *zur* regulated genes and several newly identified genes that were either repressed or activated in response to zinc (S1 Fig, R^2^ = 0.9639). Additionally, we confirmed Zur-dependent regulation by complementing the *zur* mutant and testing gene expression for eleven zinc-regulated targets by RT-qPCR (Fig 2); the eleven genes tested represent most of the zinc- and Zur-regulated genes and operons identified in *C*. *diphtheriae* (Table 1). The RT-qPCR expression data in Fig 2 largely confirms the findings in the microarray, with exception of gene *dip0092*, which was not significantly zinc-regulated in the wild-type strain using RT-qPCR (Fig 2). It is unclear why the expression of *dip0092* measured using RT-qPCR is different from that of the array, but it is likely due to differences in the methodology and the sequences of the probes. It should be noted that complementation of the *zur* deletion restored wild-type expression profiles for all of the genes tested by RT-qPCR, including *dip0092* (Fig 2).

**Table 1:** Genes differentially regulated by zinc and Zur.

Locus	Gene Product		WT ± Zn ^A^	Δ*zur*:WT^B^
*dip0013*		hypothetical protein	-1.47	1.33
*dip0092*		two-component sensor histidine kinase	-1.56	2.05
*dip0093*		putative membrane protein	1.21	4.05
*dip0173*	IutE	zinc ABC transporter substrate-binding protein	-2.00	1.12
*dip0438* ^*C*^	TroA	anchored repeat ABC transporter, substrate-binding protein	-2.84	2.87
*dip0439*		putative membrane protein	-2.54	2.71
*dip0440*		anchored repeat-type ABC transporter ATP-binding subunit	-2.42	2.65
*dip0441*		anchored repeat-type ABC transporter permease subunit	-2.43	2.95
*dip0442*		ABC transporter permease	-3.42	3.51
*dip0443*		putative surface-anchored protein	-3.18	3.3
*dip0444*		putative membrane protein	-2.9	3.38
*dip0445*		DUF3068 domain-containing protein	-1.09	1.32
*dip1087*		hypothetical protein	-1.18	1.19
*dip1101* ^*D*^	CztA	cation-efflux system integral membrane protein		5.39
*dip1486*	Zrg	GTP-binding protein	-5.43	5.45
*dip1724*	CmrA2	surface-anchored membrane protein	-5.35	5.62
*dip_RS20265*		hypothetical protein	-2.39	-1.01
*dip1834*		hypothetical protein	-2.23	-1.29
*dip2114*	AdhA	alcohol dehydrogenase	3.39	-3.32
*dip2125*		ABC transporter ATP-binding protein	-1.53	2.37
*dip2126*		peptide ABC transporter permease	-1.47	2.09
*dip2127*		ABC transporter permease	-1.67	2.23
*dip2128*		substrate-binding transport protein	-2.07	2.35
*dip_RS21920* ^*C*^		hypothetical protein	-2.1	1.27
*dip_RS21925*		hypothetical protein	-2.37	1.97
*dip2158*	CdtQ	ABC transporter ATP-binding protein	-2.29	2.36
*dip2159*	CdtP	ABC transporter ATP-binding protein	-2.82	2.89
*dip2160*	SidB	polyketide synthase	-2.53	3.05
*dip2161*	SidA	non-ribosomal peptide synthetase	-2.97	3.52
*dip2162*		ABC transporter substrate-binding protein	-1.71	1.57
*dip2163*		ABC transporter permease	-1.89	2.17
*dip2164*		ABC transporter permease	-2.03	2.28
*dip2165*		ABC transporter ATP-binding protein	-2.24	2.36
*dip2323* ^*C*^	HrtA	ABC transporter ATP-binding protein	-1.99	1.94
*dip2324*	HrtB	ABC transporter permease	-3.25	4.11
*dip2325*	CmrA	surface-anchored protein	-5.36	5.22

Log_2_ fold change indicated for all genes.

^A^ For WT ± Zn, positive values indicate increased expression with zinc supplementation; negative values indicate reduced expression with zinc supplementation.

^B^ For Δ*zur*:WT, positive values indicate higher expression in the *zur* mutant; lower values correlate with lower expression in the *zur* mutant.

^C^ Gene clusters described in the text are shaded (blue).

^D^ No significant zinc-regulation was observed for *cztA* (*dip1101*).

#### Analysis of transcriptional activity within the *troA-dip0445* gene cluster

**Fig 3:**
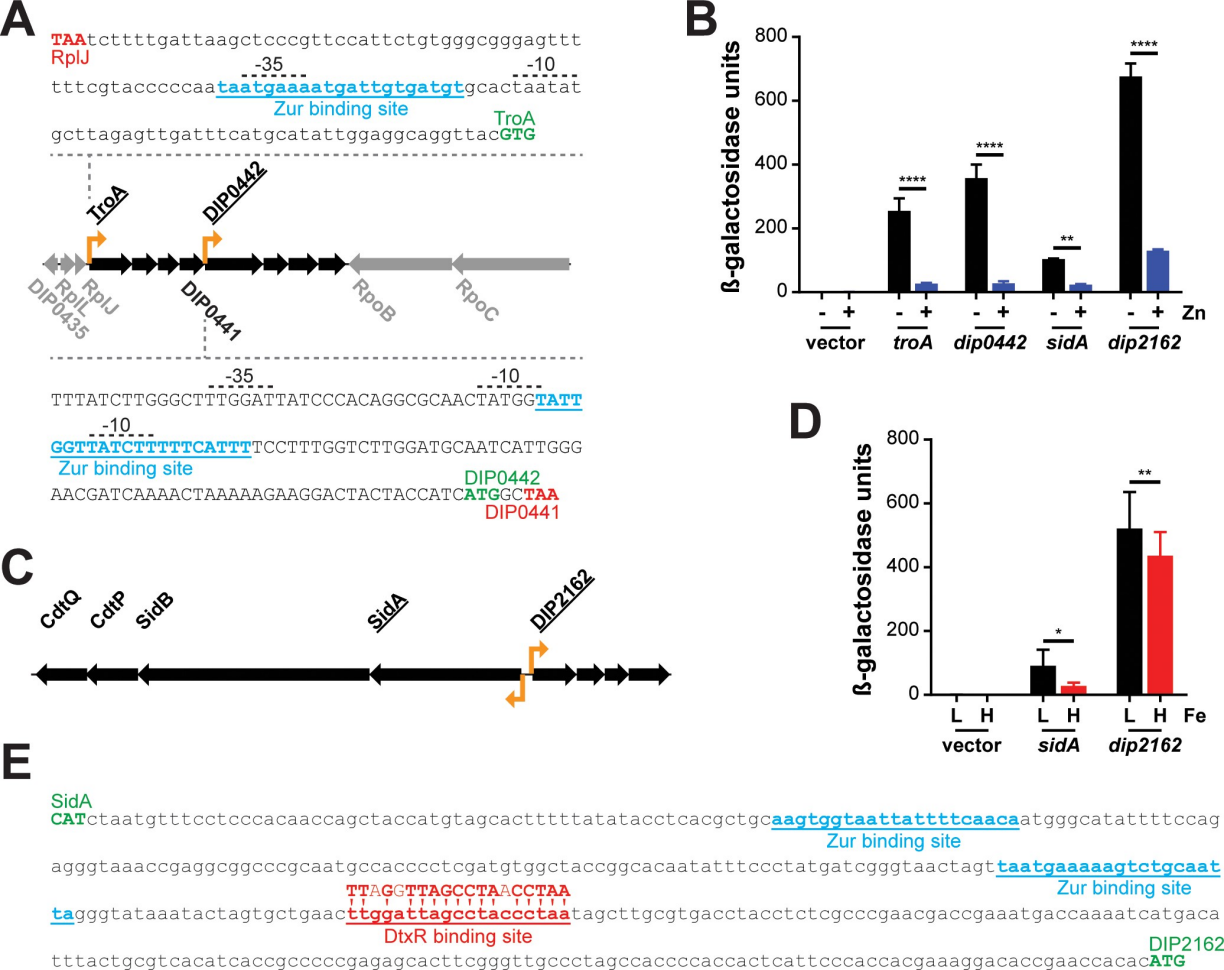
Zinc regulated promoters of *troA* (*dip0438)*, *dip0442*, *sidA* (*dip2161)*, and *dip2162*. (A) Genes of the *troA-dip0445* gene locus encode a putative ABC transport system and several membrane proteins of unknown function. Flanking genes encode ribosomal proteins and components of RNA polymerase (RpoB and RpoC) (grey). DNA sequence of the promoter regions for *troA* (top) and *dip0442* (bottom) with notable features are shown: Zur binding sites (blue), start codons (green), stop codons (red), and predicted -10 and -35 elements (promoter elements were not experimentally determined, but based on the consensus sequences determined previously [15]. Coding regions are uppercase, while non-coding regions are lowercase. (B) Promoter fusions for *troA*, *dip0442*, *sidA*, and *dip2162* were tested for activity in mPGT without (**-**) and with (**+**) zinc supplementation (5 μM), n = 3. (C) Genes of the *dip2158* (*cdtP*)*-sidA* region encode a putative yersiniabactin-transport (*cdtPQ*) and synthesis (*sidAB*) like locus that is adjacent to a putative ABC transporter (*dip2162-dip2165*). (D) Promoter fusions for *sidA* and *dip2162* were tested for activity in mPGT with low (**L**) (0.5 μM) and high (**H**) (10 μM) iron supplementation in the absence of added zinc; n = 4. The data are the mean and standard deviation of indicated biological replicates for each experiment. All data were analyzed by 2way ANOVA, Holm-Sidak: **** indicates p <0.0001, ** indicates p < 0.01, and * indicates p < 0.05. (E) DNA sequence of the *sidA-dip2162* intergenic region. Notable features are indicated: Zur binding sites (blue), DtxR binding site (red) with consensus DtxR binding site above (red) and start codons (green).

We previously showed that the *C*. *diphtheriae troA* gene (*dip0438*) was transcriptionally regulated by zinc and Zur, but the regulation of downstream genes in this large genetic cluster (*dip0438-dip0445*) was not determined [13]. In our gene expression analysis, we observed a difference in the level of zinc-dependent repression between the *troA-dip0441* region and the downstream genes *dip0442-0445* (Table 1). This difference in expression was also observed when comparing the wild-type strain and *zur* mutant. We hypothesized that a second zinc-regulated promoter is present upstream of *dip0442* (Fig 3A). To test for the presence of an additional zinc-regulated promoter, we cloned the promoter region of *troA* (126-bp) and the 350-bp region upstream of *dip0442* into a *lacZ* reporter plasmid (pSPZ) to create a promoter-*lacZ* transcriptional fusion construct. These promoter-*lacZ* fusion plasmids were moved into wild-type *C*. *diphtheriae* and tested for promoter activity in mPGT in the absence and presence of zinc (Fig 3B). We observed high levels of promoter activity from both constructs in the absence of zinc supplementation, while expression from both promoters was strongly repressed in the presence of zinc. These findings suggest that both the *dip0438* and *dip0442* promoter regions may contain a Zur binding site, and the absence of an intergenic region between *dip0441* and *dip0442* indicates that the promoter for *dip0442* is within the *dip0441* coding region. The presence of a promoter in a coding region in *C*. *diphtheriae* has not been previously described.

The *troA-dip0445* gene cluster is predicted to encode an ABC type metal transporter and several surface-anchored membrane proteins of unknown function. The ABC type metal transporter is comprised of TroA, a putative substrate binding protein, and DIP0440 and DIP0441 as the ATP-binding protein and permease components, respectively. The *dip0439* gene, located between *troA* and *dip0440*, encodes a putative secreted/membrane protein of unknown function, and has sequence similarity to *dip0442*, *dip0443*, *cmrA2* (*dip1724*), and *cmrA* (*dip2325*). Although the function for the product of *dip0439* is not known, its proximity to genes encoding an ABC metal transporter suggests that it may encode a product associated with metal uptake. The products of *cmrA*, *cmrA2*, and *dip0443* are predicted to localize to the cell wall through sortase-anchoring mechanisms; however, the function of these proteins remains unclear. Many of the sortase-anchored proteins in *C*. *diphtheriae* are pilin subunits [16–18]; sortase-anchored proteins that are not associated with pilins have no known function, with the exception of DIP2093, which is proposed to function as an adhesin [19].

We further noted that the *troA-dip0445* gene cluster is flanked by ribosomal genes *rplL* and *rplJ*, and the RNA polymerase subunit genes *rpoB* and *rpoC* (Fig 3A). Zinc is an essential cofactor for RNA polymerase and is required for certain ribosomal proteins to function, and zinc starvation has been shown to induce expression of alternative ribosomal proteins in *Mycobacteria* [20]. Although the expression of these ribosomal proteins was not affected by zinc availability, it is possible that the products of these genes require zinc for function and may explain their genetic linkage to an ABC-type manganese/zinc transporter.

#### Iron and zinc regulation of *sidA* and *dip2162*

We identified a gene cluster extending from *dip2158* to *dip2165* that included two distinct operons transcribed in opposing directions (Table 1 and Fig 3C). The *cdtPQ-sidAB* and *dip2162-2165* loci are predicted to be divergently transcribed from a 387-bp region located between the two operons (Fig 3C and 3E). The genes *sidA* and *sidB*, which are predicted to encode a non-ribosomal peptide synthase and a polyketide synthase, respectively, were previously characterized as iron- and DtxR-regulated [21]. Additionally, in a recent transcriptomic analysis of *C*. *diphtheriae*, the *dip2162-2165* operon was also shown to be regulated by DtxR [15]. We cloned the 387-bp intergenic region in both orientations upstream of the *lacZ* gene in our reporter plasmid to test for differences in promoter activity in the presence and absence of zinc and iron (Fig 3B and 3D). The *sidA* promoter displayed lower overall activity than the *dip2162* promoter, but both promoters were repressed in response to zinc and iron (Fig 3B and 3D). The sequence of the 387-bp intergenic region shown in Fig 3E indicates the location of two predicted Zur binding sites and the previously reported DtxR binding site [21]. The presence of a single DtxR binding site in this region suggests that iron regulation of both *sidA* and *dip2162* is controlled by this single DtxR binding site (Fig 3D). Analysis of the sequence did not identify additional DtxR binding sites in this intergenic region

The *dip2162*-*2165* genes are predicted to encode an ABC transporter, which includes the putative substrate binding protein DIP2162, two permease proteins (DIP2163 and DIP2164), and an ATPase component (DIP2165). Interestingly, DIP2162 belongs to the NikA/OppA-like family of substrate binding proteins. A similar transporter in *S*. *aureus*, CntABCDF, was shown to have roles in zinc acquisition, and, like *dip2162-dip2165*, appears to be regulated by both iron and zinc [22]. CntABCDF functions as the transporter for staphylopine, which serves as a zincophore, a small secreted molecule that binds extracellular zinc and is subsequently imported into the bacterium. While *C*. *diphtheriae* produces the siderophore, corynebactin [23], there have been no studies or evidence for the synthesis of small molecules in *C*. *diphtheriae* that are able to bind and assist in the transport of zinc. The *sidAB* genes are predicted to encode enzymes associated with the synthesis of small molecules, although no product for these genes has been identified; it is possible that the product of the *sidAB* genes may be associated with zinc or iron metabolism. Downstream from *sidAB* are two genes, *cdtPQ*, with homology to ABC transport proteins, and the *cdtPQ-sidAB* genetic locus shares similarity to the yersiniabactin siderophore operon [21]. A recent study reported a role for yersiniabactin in zinc acquisition as a zincophore [24]. Independent of the *cdtPQ-sidAB-dip2162-2165* cluster, a separate zinc- and Zur-regulated locus, *dip2125-2128* (Table 1), also encodes an ABC transporter similar to *dip2162-dip2165*; the transporters encoded by *dip2125-2128* and *dip2162-2165* may be responsible for transporting molecules similar to yersiniabactin and staphylopine. Additional studies are needed to explore the function of these zinc-regulated transporters as well as the identification and characterization of the product of the *sidAB* genes. The regulation of *sidAB* and associated ABC transport genes by zinc and Zur suggests a potential role in zinc acquisition or metabolism.

#### Analysis of the promoter regions for *cmrA* and *hrtAB*

**Fig 4:**
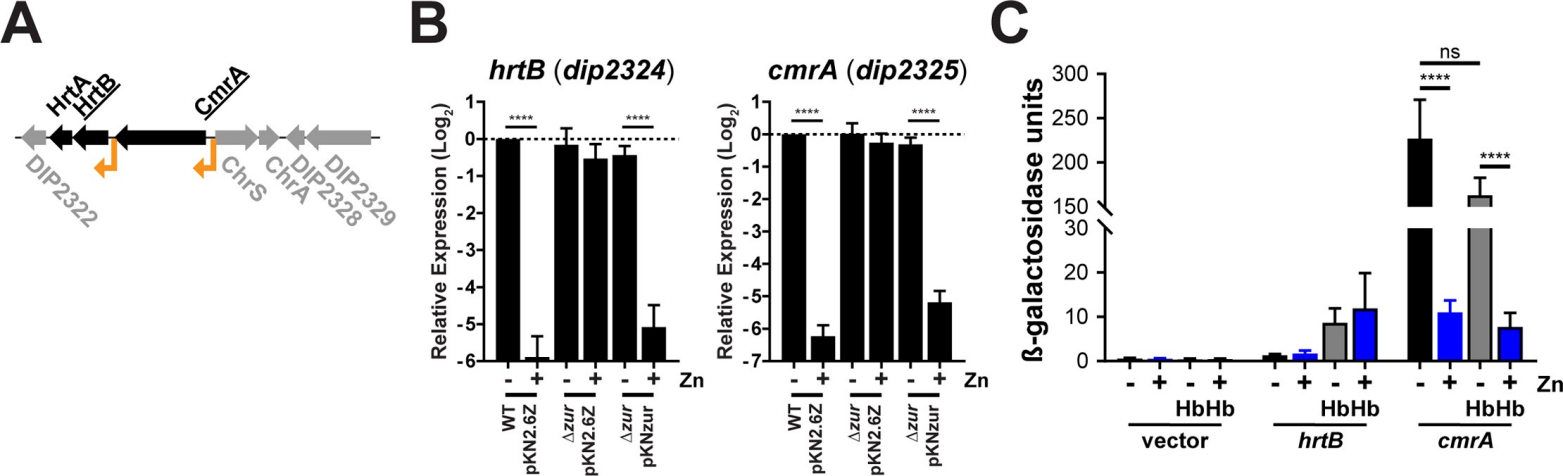
Zinc regulation of *cmrA*. (A) Genetic map of the *cmrA-hrtBA* region. The *cmrA* gene encodes a putative sortase-anchored protein; *hrtBA* encodes a heme export system. Putative promoters for both loci are indicated (arrows). (B) RT-qPCR for *hrtB* and *cmrA* in wild-type *C*. *diphtheriae* with empty vector (WT pKN2.6Z) and the Δ*zur* mutant (Δ*zur*) with empty vector (pKN2.6Z) and *zur* complementing plasmid (pKNzur). Expression of each gene was tested without (**-**) and with (**+**) 5 μM zinc supplementation. The data are the mean and standard deviation of three biological replicates. Expression of the *gyrB* gene was used for normalization; significance was determined by 2way ANOVA, Holm-Sidak’s multiple comparisons test: **** indicates p < 0.0001; * indicates p < 0.05. (C) Promoter fusions for *hrtB* and *cmrA* were tested for activity in mPGT without (**-**) and with (**+**) zinc supplementation (5 μM), and hemoglobin (**Hb**) supplementation (100 μg/ml); n = 3. The data are the mean and standard deviation of indicated biological replicates for each experiment. All data were analyzed by 2way ANOVA, Holm-Sidak: **** indicates p <0.0001, ** indicates p < 0.01, and * indicates p < 0.05.

In our gene expression analysis, we observed zinc and Zur-dependent repression of *hrtB*, *hrtA*, and *cmrA* (*dip2323*-*dip2325*) (Table 1). The *cmrA* gene encodes a putative sortase-anchored surface protein [13] and the downstream genes, *hrtBA*, encode a putative heme efflux system, which is required for survival during growth in high heme levels (Fig 4A) [25]. The promoters upstream of *cmrA* [13] and *hrtBA* [25] were characterized previously. Expression of *hrtBA* is regulated by the ChrAS two-component system in a heme or hemoglobin-dependent manner [25]. An *hrtB* promoter-*lacZ* fusion construct showed that transcriptional activity from the *hrtB* promoter was activated in the presence of hemoglobin, which was dependent on the ChrAS transcriptional regulator. Expression from the promoter upstream of the *cmrA* gene was previously reported to be strongly repressed by zinc and Zur [13]. However, we found *hrtB*, *hrtA*, and *cmrA* to be regulated by zinc in a Zur-dependent manner (Table 1). We confirmed the zinc and Zur regulation of *hrtB* and *cmrA* by RT-qPCR and showed that wild-type expression levels were restored in the *zur* mutant background by the cloned *zur* gene (Fig 4B). To determine the effect of zinc and hemoglobin on transcription at the *hrtB* and *cmrA* promoters, we compared expression from both promoters using previously described *lacZ*-promoter fusion constructs for *hrtB* [25] and *cmrA* [13]. Transcription from the *hrtB* promoter was weakly activated by the addition of hemoglobin, while zinc supplementation had no discernible effect on expression (Fig 4C). The *cmrA* promoter fusion showed strong repression by zinc, while hemoglobin had no significant effect on transcription. These results are consistent with previous findings for the promoter activities detected for *hrtB* and *cmrA*, and suggest that the zinc and Zur regulation of *hrtBA* is a result of read-through transcription from the zinc-regulated *cmrA* promoter.

### Identification and verification of newly identified Zur binding sites

**Fig 5:**
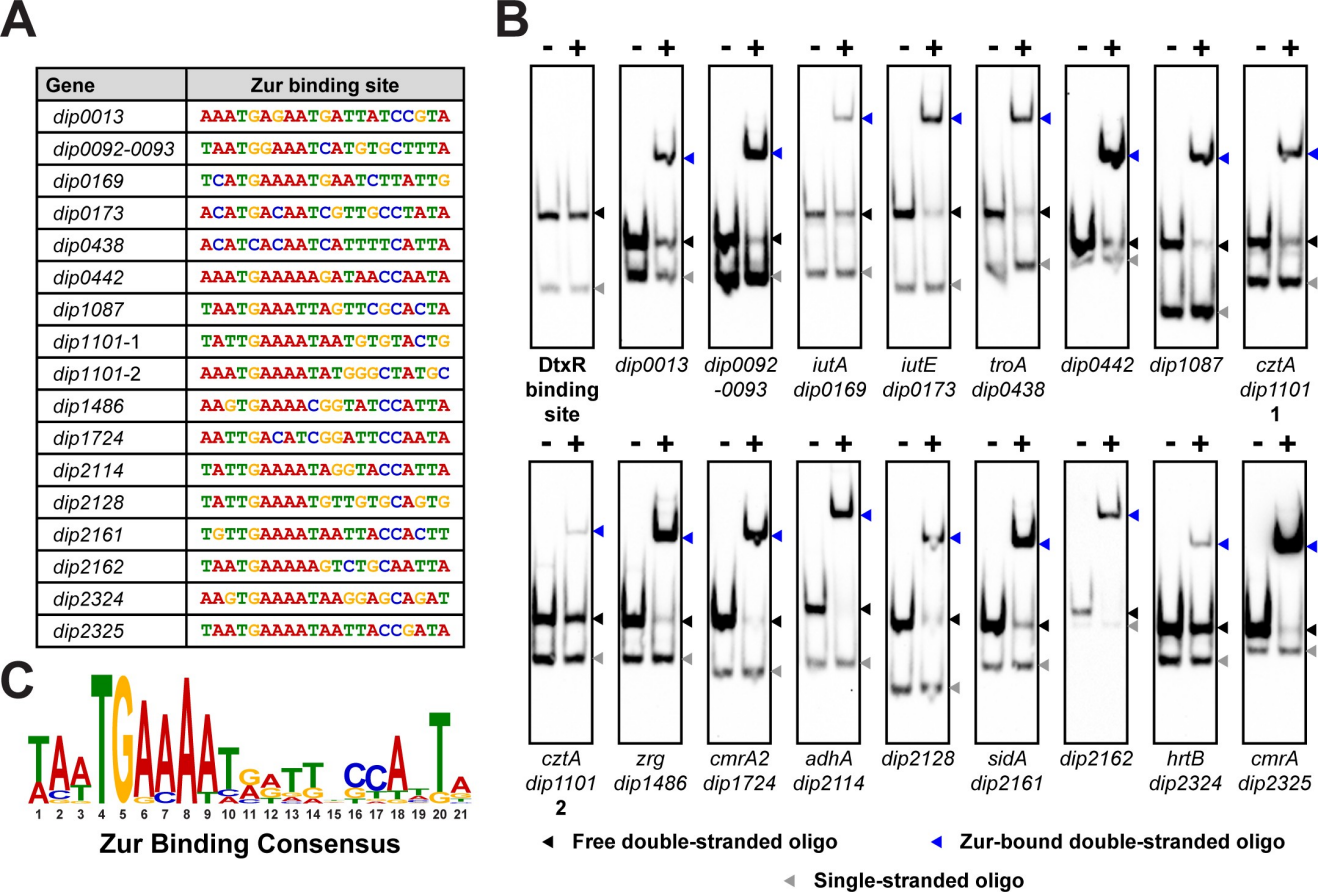
Identification of Zur binding sites. (A) Sequences for Zur binding sites predicted by MEME [26] found within putative promoter regions of indicated genes. The *cztA* gene (*dip1101)* contains two potential binding sites (1 and 2). (B) EMSAs used 100 nM recombinant Zur and 1 nM biotinylated oligos containing the putative binding sequence. (**–**) indicates lanes in which no Zur was added; (**+**) indicates the addition of His-tagged Zur protein. The DtxR binding site of *iutA* was used as a negative binding control. Arrows indicate single-stranded oligo (grey), unbound double-stranded oligo (black), and Zur-bound double-stranded oligo (blue). 5 μM zinc chloride was present in all binding reactions. The Zur binding consensus was generated from the binding sequences indicated in (A).

In a previous study of the *iut* gene cluster [8], we identified Zur binding sequences upstream of *iutA* (*dip0169*) and *iutE* (*dip0173*) using a consensus sequence generated from a study of the Zur regulon in *C*. *glutamicum* [11]. Using this consensus sequence, we employed the MEME prediction tool to search for potential Zur binding sites upstream of genes identified to be Zur-regulated in the microarray (Fig 5A). To test these putative Zur binding sites, we purified recombinant His-tagged Zur by affinity chromatography followed by dialysis in the presence of the metal chelator EDTA and Chelex 100 resin to remove metals from the purified protein. We performed EMSAs with Zur and biotinylated, annealed oligos containing the predicted binding sequences. The predicted Zur binding sites shown in Fig 5A were able to bind Zur with varying affinities (Fig 5B). The *iutA* DtxR binding site, which was used as a negative control, did not bind Zur as expected. Based on the Zur binding sequences presented in Fig 5A, a new consensus binding sequence for Zur was established (Fig 5C).

**Fig 6:**
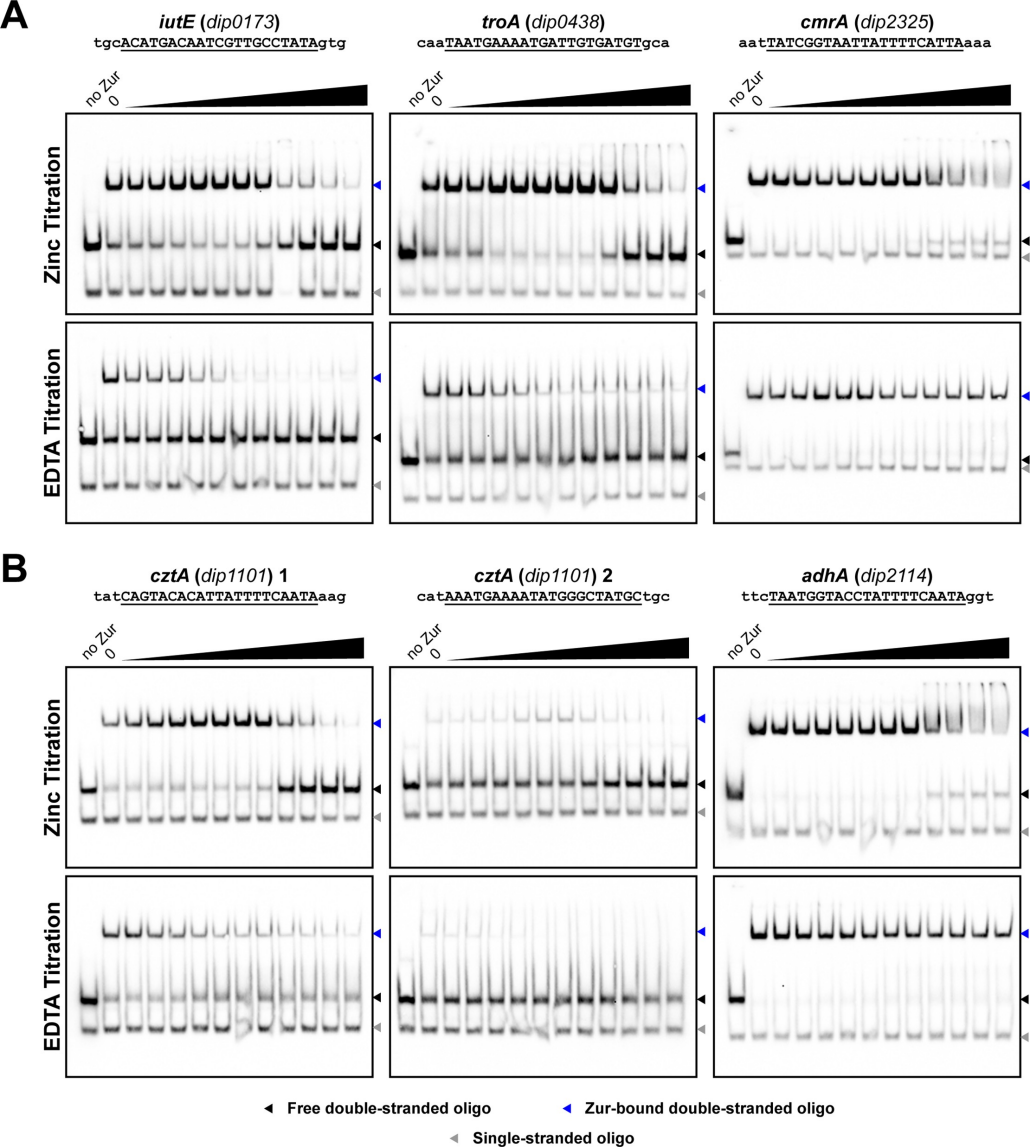
Testing metal dependence on Zur binding. Binding sites for genes which are zinc-repressed (A) and zinc-activated (B) were tested for binding in the presence of increasing concentrations of zinc or EDTA from approximately 0.25 μM to 250 μM in 2-fold increments. Binding sequences are shown with the Zur binding sequence underlined. Recombinant Zur was present at a concentration of 100 nM in all reaction conditions, except where indicated.

We also examined whether zinc is required to activate the DNA binding activity of Zur *in vitro*. We used several different Zur binding sites to test the role of zinc in Zur activation and observed that Zur was able to bind to all of the sites in the absence of added zinc (Fig 6). The Zur protein used in these studies was dialyzed in the presence of the metal chelator EDTA and Chelex 100 resin to reduce or eliminate residual zinc that may be bound to Zur. We observed that Zur binding to DNA was slightly enhanced with the addition of zinc for certain binding sites, although very high concentrations zinc appeared to inhibit DNA binding (Fig 6A and 6B). Since previous studies have shown that zinc has high affinity to Zur proteins [12], we surmised that residual zinc may remain associated with recombinant Zur despite extensive dialysis in the presence of metal chelators. To test this possibility, we examined whether Zur binding could be diminished by the addition of EDTA to the EMSA binding reactions. We observed that increasing EDTA levels reduced Zur binding at some of the DNA binding sites (Fig 6A and 6B). Probes for *adhA* and *cmrA* remained strongly Zur-associated even at the highest EDTA concentrations (250 μM), but we observed reduced Zur:DNA binding with probes for *iutA*, *troA*, and *cztA*. Taken together, the data suggest that the characteristics of Zur binding to DNA do not differ between repressed and activated genes, and the variability in binding affinity at all sites is likely due to the differences in the sequence of the binding site. Additional studies are needed to understand the mode of action of the *C*. *diphtheriae* Zur protein and in particular its interaction with zinc and the specific nature of the binding to zinc that results in DNA binding.

### CztA (DIP1101) is required for tolerance to high zinc

**Fig 7:**
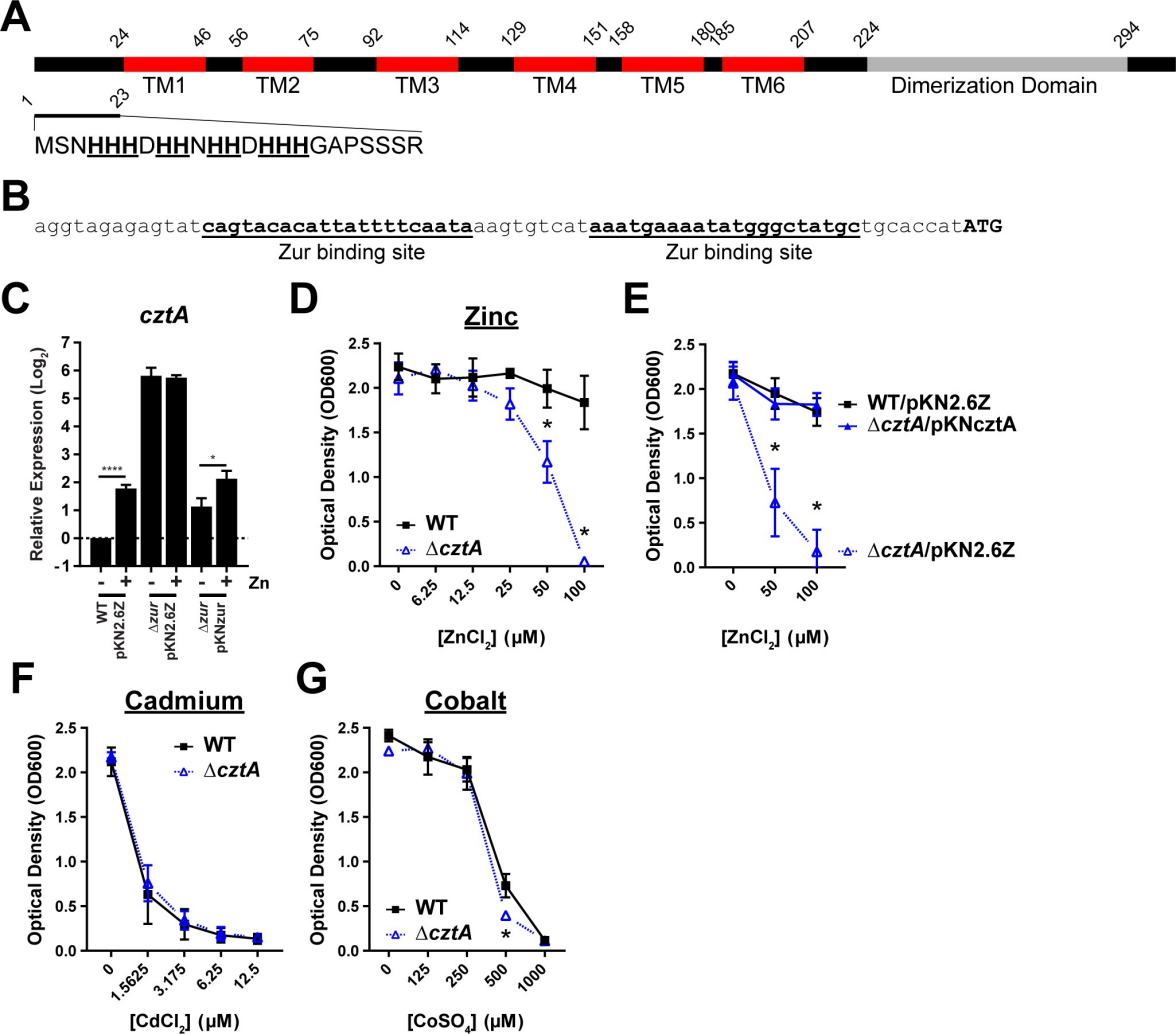
DIP1101 (CztA) encodes a membrane protein involved in zinc tolerance. (A) Structural map of the CztA protein shows multiple transmembrane domains (TM1-6), as predicted by TMHMM [27], and a dimerization domain. N-terminal amino acids 1 to 23 with histidine residues underlined; amino acids 18 to 294 share similarity with proteins of the COG1230 family of cation efflux membrane proteins. (B) Locations of verified Zur binding sites relative to the start codon (ATG) of CztA. (C) Expression of *cztA* tested by RT-qPCR in wild-type *C*. *diphtheriae* carrying vector pKN2.6Z (WT pKN2.6Z) and the *zur* mutant with vector pKN2.6Z (Δ pKN2.6Z) or the *zur* complementing plasmid, pKNzur (Δ pKNzur) without (**-**) or with (**+**) zinc supplementation. The data are the mean and standard deviation of three biological replicates. Expression of the *gyrB* gene was used for normalization; significance was determined by 2way ANOVA, Holm-Sidak’s multiple comparisons test: **** indicates p < 0.0001; * indicates p < 0.05. (D) Growth of *C*. *diphtheriae* wild type (WT) and *cztA* mutant (Δ*cztA*) in HIBTW with zinc chloride supplementation as indicated. (E) Growth of wild-type *C*. *diphtheriae* with empty vector (WT/pKN2.6Z) and the *cztA* mutant with empty vector (Δ*cztA*/pKN2.6Z) or *cztA* complementing plasmid (Δ*cztA*/pKNcztA). Growth of the wild type and *cztA* were also tested with (F) cadmium chloride and (G) cobalt sulfate. The data for growth assays are the mean and standard deviation of at least three biological replicates. * indicates significance by t-test comparing the respective wild type and mutant; p < 0.01.

The product of *cztA* is predicted to encode a cation-efflux system of the COG1230 family (Fig 7A). Proteins of this family have roles in cobalt, zinc, and cadmium efflux. In evaluating global gene expression in response to zinc and the deletion of the *zur* gene, we observed that expression of *cztA* was significantly greater in the *zur* mutant than in the wild-type strain, but we did not detect significant changes in *cztA* expression in response to zinc (Table 1). However, we verified two separate Zur binding sites (Figs 5B, and 6B) within the promoter region of *cztA* (Fig 7B) and detected weak zinc-activation by RT-qPCR in both the wild-type carrying the empty vector and the complemented *zur* mutant (Fig 7C). We observed greater expression of the *cztA* gene in the *zur* mutant, both when measuring global gene expression (Table 1), and expression by RT-qPCR (Fig 7C). While the EMSA and RT-qPCR results suggest that Zur has a direct role in regulation of *cztA* expression, the findings are not consistent with a zinc- and Zur-activated gene. In contrast, *adhA* displays the expected gene expression profile for a zinc- and Zur-activated gene (Table 1 and Fig 2); increased expression in high zinc in the wild-type strain, with comparatively low levels of expression in the *zur* mutant. The reason for increased expression of *cztA* in the *zur* mutant is unclear, and additional studies are required to understand the unusual transcriptional regulation of *cztA*.

Since CztA has sequence similarity to proteins involved in metal efflux, we generated a *cztA* deletion mutant to test the role of CztA in tolerating high-metal environments. Since proteins with homology to CztA are known to efflux cadmium, cobalt, and/or zinc, we challenged the wild-type strain and the *cztA* mutant with varying levels of each metal in HIBTW medium. Each strain was grown to mid-logarithmic phase and diluted 1:1 with media containing each respective metal to reach the final concentrations indicated. The *C*. *diphtheriae cztA* mutant showed greater sensitivity to zinc than the wild-type strain at concentrations of 25 μM and higher (Fig 7D); this phenotype was complemented by the addition of a plasmid-encoding *cztA* (Fig 7E). The wild type and mutant showed similar sensitivities to both cadmium and cobalt (Fig 7F and 7E). The data indicates that CztA contributes to zinc tolerance, but has no role in protection from toxic levels of cadmium or cobalt.

While it is well established that the host S100 proteins sequester zinc from sites of inflammation [28, 29], recent studies in *Mycobacterium tuberculosis* [10] and *Streptococcus pyogenes* [30, 31] have demonstrated important roles for zinc intoxication as a host mechanism for eliminating pathogens. In *M*. *tuberculosis*, CtpC, a P_1_-Type ATPase, is important in protecting the bacterium from high levels of zinc found within the macrophage phagosome [10]. For *S*. *pyogenes*, the CzcD protein is also a zinc specific efflux system that protects from high levels of zinc and contributes to survival against neutrophil killing and virulence in a mouse infection model [30]. Intracellular mechanisms of zinc toxicity likely stem from the mis-metalation of enzymes [9]; moreover, high extracellular levels of zinc may interfere with the transport of other metals as evidenced in *Streptococcus pneumoniae* [32]. While it is unclear where *C*. *diphtheriae* might encounter zinc intoxication within the host, it is likely that damage to host tissue results in the recruitment of immune cells to the site of both respiratory and cutaneous infections. We have a limited knowledge of *C*. *diphtheriae* interactions with the host immune system and the mucosal surfaces of the upper respiratory tract [33], and additional studies are necessary to determine whether *C*. *diphtheriae* encounters toxic zinc levels within the human host.

## Materials and Methods

### Bacterial strains and media

Bacterial strains and plasmids used in this study are listed in Table 2. *C*. *diphtheriae* strains were routinely grown in heart infusion broth with 0.2% (v/v) Tween 80 (HIBTW) or on heart infusion agar (1.5% w/v) at 37°C. *E*. *coli* strains were grown in Luria-Bertani (LB) medium or on LB agar (1.5% w/v). Strains were stored at -80°C in their respective culture media with 20% v/v glycerol. mPGT medium containing 0.5% w/v Casamino Acids treated with Chelex100 and supplemented with 0.5 μM FeCl_3_ was used for testing gene expression; high-iron mPGT included 10 μM FeCl_3_. 5 μM ZnCl_2_ or 100 μg/ml hemoglobin (MP Biomedicals) was used where indicated. Antibiotics were used at 50 μg/ml for kanamycin, 100 μg/ml for spectinomycin, and 10 μg/ml for nalidixic acid.

**Table 2:** Strains and Plasmids.

Strain or plasmid	Description or use	Reference or source
*C*. *diphtheriae* strains		
1737	Wild type, Gravis biotype, Tox^+^	[34]
1737 Δ*zur*	Deletion of *zur* in 1737	[8]
1737 Δ*cztA*	Deletion of *cztA* in 1737	This study
*E*. *coli* strains		
NEB 5-alpha competent	Cloning strain	New England Biolabs, Inc.
BL21(DE3)	Protein expression	[35]
S17-1 λpir	Mating strain	[36]
Plasmids		
pET30zur	*C*. *diphtheriae zur* cloned into pET30a	[8]
pKN2.6Z	*C*. *diphtheriae* shuttle vector, Kn^r^	[37]
pKNzur	pKN2.6Z carrying the *C*. *diphtheriae zur* gene	This study
pKNcztA	pKN2.6Z carrying the *C*. *diphtheriae cztA* gene (*dip1101*)	This study
pSPZ	Carries *lacZ*; Spc^r^	[38]
pSPZ-dip0438	126 bp upstream of *dip0438* fused to *lacZ* in pSPZ	This study
pSPZ-dip0442	350 bp upstream of *dip0442* fused to *lacZ* in pSPZ	This study
pSPZ-dip2161	387 bp intergenic region upstream of *dip2161* fused to *lacZ* in pSPZ	This study
pSPZ-dip2162	387 bp intergenic region upstream of *dip2162* fused to *lacZ* in pSPZ	This study
pSPZ*cmrA*	*cmrA* promoter-*lacZ* fusion in pSPZ	[13]
phrtB-lacZ	*hrtB* promoter-*lac*Z fusion in pSPZ	[25]
pKΔ1101	Suicide vector for deletion of *dip1101*(*cztA*)	This study

### RNA extraction

*C*. *diphtheriae* strains were grown overnight in mPGT with 1 μM FeCl_3_ and antibiotics as needed. Overnight cultures of *C*. *diphtheriae* were diluted into mPGT with 1 μM FeCl_3_ with antibiotics as needed and grown at 37°C for 2 hrs. Cells were then diluted to a final OD600 of 0.09 in mPGT with 0.5 μM FeCl_3_ without or with 5 μM ZnCl_2_ or antibiotic supplementation and grown to logarithmic phase. The *zur* mutant was only grown in high zinc (5 μM) for the array. Cultures were mixed with 95% EtOH with 5% phenol on ice and cells were collected through centrifugation. Cells were lysed by mechanical lysis with Lysing Matrix B (MP Biomedicals) in PBS with 9.5% EtOH, 0.5% phenol, and 14.3 mM β-mercaptoethanol. TRIzol LS reagent (Thermo Fisher Scientific) was added to cell lysates and RNA was isolated following the Direct-zol RNA (Zymo Research) extraction protocol. RNA was treated with TURBO DNA-free Kit (Ambion, Life Technologies) following the extended treatment protocol. RNA integrity was assessed using an Agilent RNA 6000 Nano chip and Bioanalyzer.

### Agilent single-channel microarray preparation, data collection, and analysis

Custom microarrays (8 microarrays per slide) based on the published *C*. *diphtheriae* NCTC 13129 genome [39] were designed using Agilent’s eArray platform. The Agilent One-Color Microarray-Based Exon Analysis, Version 2.0 protocol was followed as per manufacturer directions. The random oligo primer mix was used for cDNA synthesis and Cyanine 3-CTP was used for subsequent cRNA synthesis. Total RNA collected from 3 independent biological replicates of each strain and condition was used for the synthesis of cRNA. Each sample was processed and run in individual wells. Arrays were washed as directed and scanned using an Agilent G2505C Microarray Scanner. For each well, the median of the gProcessedSignal for each gene probe was normalized by the mean of the entire sample set. Normalized values were log2 transformed and subjected to the J5 test using the CBER High-Performance Integrated Virtual Environment (HIVE) [40]. Genes with a log2 fold change greater than 1 and a J5 test value greater than 2 were considered significantly regulated. The microarray data and design are available at the Gene Expression Omnibus database (Accession: GSE131485).

### cDNA synthesis and qPCR

The ProtoScript II First Strand cDNA synthesis kit (New England Biolabs, Inc.) was used to synthesize cDNA from 1 μg of total RNA. qPCR was performed using the LightCycler 96 (Roche) and Luna Universal qPCR master mix (New England Biolabs, Inc.). Primers used for qPCR were designed using Primer3 and are listed in S1 Table. Data were analyzed using the ΔΔCq method and significance determined using 2way ANOVA, Holm-Sidak’s multiple comparisons test on ΔCq values. ΔCq values for *gyrB* were used for normalization. The expression of *gyrB* (*dip0005*) was consistent across conditions in our analysis.

### Plasmid construction

DNA inserts that were ligated into plasmids were initially amplified by PCR from wild-type *C*. *diphtheriae* strain 1737 genomic DNA. Primers for cloning are listed in S2 Table. The NEBuilder HiFi DNA assembly cloning kit (New England Biolabs, Inc.) was used to insert amplified DNA into pSPZ, pKN2.6Z, and pK18mobsacB.

### Beta-galactosidase assays

Overnight cultures of *C*. *diphtheriae* with indicated plasmids were grown in mPGT with 1 μM FeCl_3_. Cultures were diluted 1:1 with fresh media and grown for 4–6 h. Cultures were then diluted to a final OD600 of 0.045 in fresh media with indicated metal supplementation. Following overnight growth, cells were pelleted and treated with 10 mg/ml lysozyme in PBS at 37°C for 30 min. Following lysozyme treatment, beta-galactosidase activity assays were performed as described by Miller [41]. At least three biological replicates were tested for each plasmid and condition.

### Purification of recombinant His-tagged Zur protein

Recombinant His-tagged Zur was purified following induction in BL21(DE3) as described previously [8]. Following affinity purification, purified protein was dialyzed overnight in PBS with 1 mM EDTA followed by dialysis against PBS with 2 g/l Chelex100 for 4 h. A final dialysis was performed against PBS with 15% (v/v) glycerol with 2 g/l Chelex for 4 h. Protein was aliquoted and stored at -80°C. Protein was characterized by SDS-PAGE and analyzed an Agilent Protein 230 chip and Bioanalyzer. Purified protein concentration (nM) was determined based on the predicted molecular weight of 20.9 kD for the recombinant protein and protein concentrations (ng/μl) were determined by a Bioanalyzer.

### Electrophoretic mobility shift assays

For Zur binding assays, complementary oligos (S3 Table) containing the putative binding sequence were annealed by mixing equimolar amounts and denaturation for 2 h at 98°C in a thermocycler. The temperature was reduced from 98°C to 4°C in 1 min intervals; annealed oligomers were stored at -20°C until use. Binding reactions were performed as described previously [8] with modifications; binding was performed using 100 nM recombinant His-tagged Zur and 1 nM of annealed oligomers in 10 mM Tris, pH 7.5, 55 mM KCl, 1 mM DTT, 10% glycerol, 0.05% NP-40, 100 μg/ml BSA, and 0.03 μg/ml salmon sperm DNA. For assessing binding, 5 μM ZnCl_2_ was added to all reactions. For testing the impact of zinc and EDTA on binding, a final concentration 250 μM ZnCl_2_ or EDTA and 2-fold dilutions down to approximately 0.25 μM were tested. Reactions were separated by gel electrophoresis (10% acrylamide with 45 mM Tris-borate [0.5x TB]), and transferred onto a nylon membrane in 0.5x TBE (45 mM Tris-borate, 1 mM EDTA). Biotinylated oligos were detected using the LightShift chemiluminescent EMSA kit (Thermo Scientific).

### Generation of a *C*. *diphtheriae dip1101* deletion mutant

Mutation of *cztA* (*dip1101*) in *C*. *diphtheriae* was introduced by allelic exchange using *E*. *coli* strain S17-1 λpir for conjugation [18] and the plasmid pKΔ1101 generated from pk18mobsacB, which encodes for kanamycin resistance and sucrose sensitivity. pKΔ1101 introduces an in-frame deletion of *cztA*, removing most of the coding region and results in a 14 amino acid product. *C*. *diphtheriae* that had integrated the plasmid were isolated on HIA with nalidixic acid and kanamycin. Subsequently, HIA with 10% sucrose was used to isolate bacteria in which a second recombination event had occurred. Isolates were tested for sucrose resistance and kanamycin sensitivity. Deletion of the *cztA* locus was confirmed by PCR using primers external to the deletion construct.

### Metal tolerance growth assays

Overnight HIBTW cultures of *C*. *diphtheriae* wild type or the *cztA* mutant were diluted 1:1 with fresh media and grown for 4–6 h. Cultures were then diluted into HIBTW to an OD600 of 0.09 and diluted 1:1 into HIBTW with CdCl_2_, CoSO_4_, or ZnCl_2_ supplementation for a starting OD600 of 0.045 and final metal concentrations as indicated. OD600 was measured after overnight growth. Kanamycin was added for complementation experiments.

### Statistical data analysis

GraphPad Prism version 7.0 was used for statistical analysis. Specific tests used, and P values are indicated in figure legends.

## Supporting information

S1 Fig Array correlation and complementation. (A) Selected genes were tested for expression by RT-qPCR using the same RNA samples processed for the microarray comparing the wild type grown in media without and with zinc supplementation. (B) Log_2_ fold change detected by RT-qPCR and microarray were compared with R^2^ value indicated.
(DOCX)

S1 Table Primers used for qPCR. (DOCX)

S2 Table Primers used for cloning. (DOCX)

S3 Table Primers used for EMSA. (DOCX)
