# Supplementary material for: Identification of zinc and Zur-regulated genes in Corynebacterium diphtheriae
Source: PLoS One. 2019 Aug 27;14(8):e0221711. doi: 10.1371/journal.pone.0221711 (PMC6711530; doi:10.1371/journal.pone.0221711)
Supplement: S1 Fig — (A) Selected genes were tested for expression by RT-qPCR using the same RNA samples processed for the microarray comparing the wild type grown in media without and with zinc supplementation. (B) Log2 fold change detected by RT-qPCR and microarray were compared with R2 value indicated. (DOCX) [file pone.0221711.s001.docx]

**
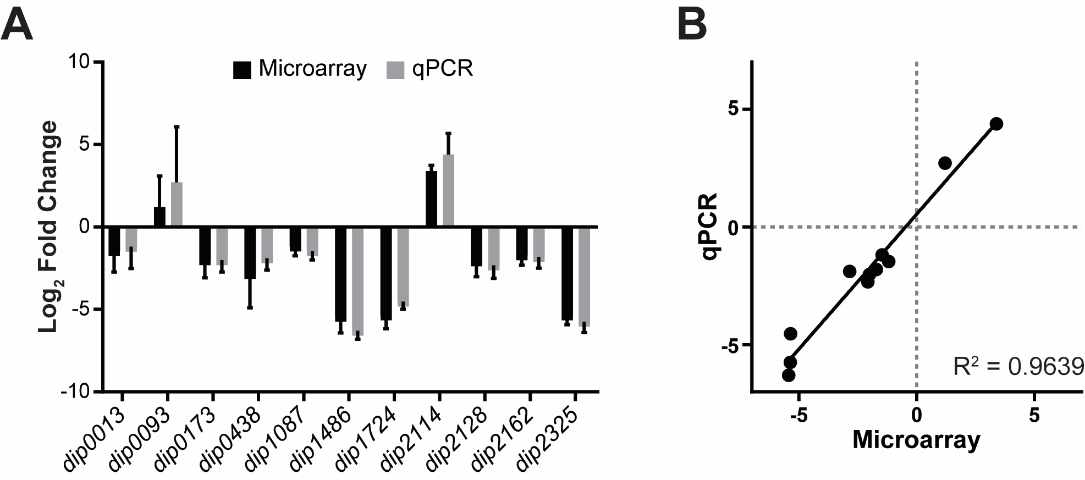
**

**S1 Fig. Array correlation and complementation.** (A) Selected genes were tested for expression by qPCR using the same RNA samples processed for the microarray comparing the wild type grown in media without and with zinc supplementation. (B) Log_2_ fold change detected by qPCR and microarray were compared with R^2^ value indicated.
