# Supplementary material for: Identification of zinc and Zur-regulated genes in Corynebacterium diphtheriae
Source: PLoS One. 2019 Aug 27;14(8):e0221711. doi: 10.1371/journal.pone.0221711 (PMC6711530; doi:10.1371/journal.pone.0221711)
Supplement: S1 Table — (DOCX) [file pone.0221711.s002.docx]

**S1 Table. Primers Used for qPCR.**

| **Gene** | **Primer Sequence (5’-3’)** | **Amplicon Size (bp)** |
| --- | --- | --- |
| *dip0005* | GGTCTGACCATTACGCTGGT | 166 |
|  | TCTTCTCGCGTTTCTTTGGT |  |
| *dip0013* | ACACCAGAAGAGGGAGAGCA | 153 |
|  | TGGGATGTCAAATTCGGAGT |  |
| *dip0092* | ACCACTACCATTGCCACCAT | 169 |
|  | ATCGCCAGTGCCAGATAAAA |  |
| *dip0093* | TGGCCATCGTGAATGTAATC | 250 |
|  | TGACCCACGTAGTTCACGAG |  |
| *dip0173* | ACCACGTCCCAGTGATCTTC | 171 |
|  | ACTTCAGGGCACCGATGTAG |  |
| *dip0438* | CGCAATACCATCAATGTTGC | 162 |
|  | GGTGGTTGCTGGATCGTAGT |  |
| *dip1087* | TGCTCGGTTCGTTTTTCTTT | 153 |
|  | GTTTGTCGTCCAAGGCGTAT |  |
| *dip1101* | CCATCACCATGACCACCATA | 182 |
|  | CCGTGGAGTCTGACAGCATA |  |
| *dip1486* | GAGAAGTTGCTGGGCTTGAG | 176 |
|  | CCAAAGGACACTGCTGGATT |  |
| *dip1724* | CGCACTAAAGTGCATGTGGA | 213 |
|  | GCCGTAAAGTCGAGTTCCTG |  |
| *dip2114* | ACGACGTTGACCTTCCAGAG | 225 |
|  | CTGACCACAGCCAGACGTTA |  |
| *dip2128* | CGAAAAGCTGGTGATGGATT | 175 |
|  | CGGTCCACTCTGTAGCATCA |  |
| *dip2162* | ACCAGTGGAAAAGCAGCAAC | 176 |
|  | ACGCTCTTGGCTAACTGGAA |  |
| *dip2324* | GTTTTTAGGAATCCGCGACA | 210 |
|  | TCCGAGGAAGTGAACTCTGG |  |
| *dip2325* | CTCCAAAGCGGAAGAGATTG | 215 |
|  | CACCGGTATCTTCGGTGTCT |  |
