# Supplementary material for: Identification of zinc and Zur-regulated genes in Corynebacterium diphtheriae
Source: PLoS One. 2019 Aug 27;14(8):e0221711. doi: 10.1371/journal.pone.0221711 (PMC6711530; doi:10.1371/journal.pone.0221711)
Supplement: S2 Table — (DOCX) [file pone.0221711.s003.docx]

**S2 Table. Primers Used for Cloning.**

| **Amplicon** | **Purpose** | **Primer Sequence (5’-3’)** |
| --- | --- | --- |
| *dip1710* locus | Complementing clone | CAAAAGCTGGTACCGGGCCATATTTTCAATTCAACACCGTTAACAC |
|  |  | CGGCCGCTCTAGAACTAGTGTTATTCGGTTGCTGCAGAGCAG |
| *dip1101* locus | Complementing clone | CAAAAGCTGGTACCGGGCCTTTTGGATTTTAGGGGGTTAAGG |
|  |  | CGGCCGCTCTAGAACTAGTGCACTAATGTTGGCAAGGTAACTC |
| pKN2.6z | Vector amplification | GGCCCGGTACCAGCTTTTG |
|  |  | CACTAGTTCTAGAGCGGCCG |
| *dip0438* promoter | Promoter fusion | GATCTCCATGGACGCGTGATCTTTTGATTAAGCTCCCGTTCC |
|  |  | GGCCAGTGAATTCCCTTTCATGTAACCTGCCTCCAATATGCAT |
| *dip0442* promoter | Promoter fusion | GATCTCCATGGACGCGTGATGAAACCGCGCGCACCATG |
|  |  | GGCCAGTGAATTCCCTTTCATGATGGTAGTAGTCCTTCTTTTTAGT |
| *dip2161*  promoter | Promoter fusion | GATCTCCATGGACGCGTGAGTGTGGTTCGGTGTCCTTTC |
|  |  | GGCCAGTGAATTCCCTTTCATCTAATGTTTCCTCCCACAACCA |
| *dip2162*  promoter | Promoter fusion | GATCTCCATGGACGCGTGACTAATGTTTCCTCCCACAACCA |
|  |  | GGCCAGTGAATTCCCTTTCATGTGTGGTTCGGTGTCCTTTC |
| pSPZ | Vector amplification | ATGAAAGGGAATTCACTGGCC |
|  |  | TCACGCGTCCATGGAGATC |
| Δ*dip1101* 5’ flank | Deletion of *dip1101* locus | GAATTCGAGCTCGGTACCCCCTGCTGTCAATATTACTGTTGG |
|  |  | CAGCATAGCCCATATTTTCATTTATG |
| Δ*dip1101*  3’ flank |  | CATAAATGAAAATATGGGCTATGCTGCATGAGTTACCTTGCCAACATTAG |
|  |  | TCGACTCTAGAGGATCCCCGCAGCCCACTGGAGTTTGC |
