# Supplementary material for: Identification of zinc and Zur-regulated genes in Corynebacterium diphtheriae
Source: PLoS One. 2019 Aug 27;14(8):e0221711. doi: 10.1371/journal.pone.0221711 (PMC6711530; doi:10.1371/journal.pone.0221711)
Supplement: S3 Table — (DOCX) [file pone.0221711.s004.docx]

**S3 Table. Primers used for EMSA.**

| **Gene Locus** | **Primer Sequence (5’-3’) ^A^** |
| --- | --- |
| *dip0013* | **B-**CCCTTGAAATGAGAATGATTATCCGTATGGGGG |
|  | CCCCCATACGGATAATCATTCTCATTTCAAGGG |
| *dip0092-0093* | **B-**CCCTACTAAAGCACATGATTTCCATTAATGGGG |
|  | CCCCATTAATGGAAATCATGTGCTTTAGTAGGG |
| *dip0169* | **B-**GGGTCGCAATAAGATTCATTTTCATGACTACCC |
|  | GGGTAGTCATGAAAATGAATCTTATTGCGACCC |
| *dip0173* | **B-**CCCTGCACATGACAATCGTTGCCTATAGTGGGG |
|  | CCCCACTATAGGCAACGATTGTCATGTGCAGGG |
| *dip0438* | **B-**CCCCAATAATGAAAATGATTGTGATGTGCAGGG |
|  | CCCTGCACATCACAATCATTTTCATTATTGGGG |
| *dip0442* | **B-**CCCTGGTATTGGTTATCTTTTTCATTTTCCGGG |
|  | CCCGGAAAATGAAAAAGATAACCAATACCAGGG |
| *dip1087* | **B-**CCCGTTTAGTGCGAACTAATTTCATTATGCGGG |
|  | CCCGCATAATGAAATTAGTTCGCACTAAACGGG |
| *dip1101-1* | **B-**GGGTATCAGTACACATTATTTTCAATAAAGCCC |
|  | GGGCTTTATTGAAAATAATGTGTACTGATACCC |
| *dip1101-2* | **B-**GGGCATAAATGAAAATATGGGCTATGCTGCCCC |
|  | GGGGCAGCATAGCCCATATTTTCATTTATGCCC |
| *dip1486* | **B-**CCCAATAAGTGAAAACGGTATCCATTAAGGGGG |
|  | CCCCCTTAATGGATACCGTTTTCACTTATTGGG |
| *dip1724* | **B-**CCCCCCTATTGGAATCCGATGTCAATTAACGGG |
|  | CCCGTTAATTGACATCGGATTCCAATAGGGGGG |
| *dip2114* | **B-**CCCTTCTAATGGTACCTATTTTCAATAGGTGGG |
|  | CCCACCTATTGAAAATAGGTACCATTAGAAGGG |
| *dip2128* | **B-**CCCCGTTATTGAAAATGTTGTGCAGTGGTGGGG |
|  | CCCCACCACTGCACAACATTTTCAATAACGGGG |
| *dip2161* | **B-**CCCTGCAAGTGGTAATTATTTTCAACAATGGGG |
|  | CCCCATTGTTGAAAATAATTACCACTTGCAGGG |
| *dip2162* | **B-**GGGCCCTAATTGCAGACTTTTTCATTAACTCCC |
|  | GGGAGTTAATGAAAAAGTCTGCAATTAGGGCCC |
| *dip2324* | **B-**CCCTAGAAGTGAAAATAAGGAGCAGATCATGGG |
|  | CCCATGATCTGCTCCTTATTTTCACTTCTAGGG |
| *dip2325* | **B-**CCCAATTATCGGTAATTATTTTCATTAAAAGGG |
|  | CCCTTTTAATGAAAATAATTACCGATAATTGGG |
| *dip0169* DtxR binding site | **B-**CCCTGTTTTAGCTTAGCCCTAGCTAATTTGGGG |
|  | CCCCAAATTAGCTAGGGCTAAGCTAAAACAGGG |

^A^ **B-** denotes biotinylation of the primer for detection purposes.
